# Impact of probiotic-enriched enteral nutrition combined with an ERAS protocol on postoperative recovery and metabolic rehabilitation in laryngeal cancer patients: a single-center retrospective cohort study

**DOI:** 10.3389/fcimb.2025.1692767

**Published:** 2025-11-03

**Authors:** Xueqiong Wei, Xiaodong Zhang, Junna Zeng, Shannan Chen, Jun Liao

**Affiliations:** Department of Otolaryngology-Head and Neck Surgery, Quanzhou First Hospital Affiliated to Fujian Medical University, Quanzhou, Fujian, China

**Keywords:** laryngeal cancer, probiotics, enhanced recovery after surgery, enteral nutrition, postoperative gastrointestinal function

## Abstract

**Background:**

Postoperative patients with laryngeal cancer frequently experience nutritional imbalance and complications due to restricted oral intake and inflammatory stress, and a single ERAS or nutritional strategy is insufficient for comprehensive recovery.

**Objective:**

To evaluate the effect of probiotic-enhanced enteral nutrition combined with an ERAS nursing pathway on postoperative gastrointestinal function, inflammatory-nutritional response, and medical resource utilization in patients with laryngeal cancer.

**Methods:**

A total of 312 single-center laryngeal cancer cases from 2021–2024 were retrospectively enrolled. After 1:1 propensity-score matching, the experimental group (n = 132, probiotics + ERAS) was compared with the control group (n = 132, routine care). The primary outcome was time to first flatus. Secondary outcomes included inflammatory-nutritional indices, complications, hospital resources, and readmission. Statistical analyses used the Cox model, linear mixed-effects model, robust variance Poisson regression, Gamma-GLM, and Pearson correlation.

**Results:**

On postoperative days 1-3, energy and protein intake were significantly higher in the experimental group than in controls (both P< 0.001); a significant group × time interaction for 7-day cumulative target attainment was also observed (P< 0.001). The probability of first flatus increased by 98% (HR = 1.98 [1.55–2.52], P< 0.001). Within 72 h, rises in CRP, IL-6, and leukocyte count and declines in albumin and prealbumin were all significantly smaller in the experimental group (group × time interactions, all P< 0.001). Risks of Clavien-Dindo grade ≥ II complications, pneumonia, wound infection, and pharyngocutaneous fistula were markedly reduced (RR 0.25–0.39, P< 0.05). Median postoperative length of stay and antibiotic days fell by 3.21 and 2.48 days, respectively; cost ratio was 0.83 (0.79–0.88); 30-day readmission OR was 0.32 (0.12–0.83). ERAS adherence correlated inversely with length of stay (r = −0.59; β = −0.017; P< 0.001).

**Conclusion:**

Probiotic-enhanced enteral nutrition combined with ERAS accelerates gastrointestinal recovery, suppresses inflammation, maintains nutritional status, and significantly reduces complications and healthcare burden in the perioperative period of laryngeal cancer, supporting the integrated “micro-ecology-nutrition-process” model as an effective strategy for rapid recovery in head-and-neck surgery.

## 1 Introduction

Laryngeal cancer is one of the most common head and neck malignancies, with approximately 200,000 new cases worldwide each year, and the standard treatment centers on surgical resection (Sung et al., 2021 and Hut et al., 2025). Postoperative patients, owing to pharyngolaryngeal anatomical disruption, hypermetabolic stress, and feeding difficulties, have a malnutrition rate as high as 60%, which is closely associated with infection, wound complications, and reduced survival (Dai et al., 2024). Over the past decade, the concept of Enhanced Recovery After Surgery (ERAS) has achieved definite results in gastrointestinal and urological fields, and head and neck surgery has likewise begun to introduce measures such as goal-directed fluid therapy and multimodal analgesia to shorten hospital stay (Wishahi et al., 2024; Bertazzoni et al., 2022). Parallel studies have shown that probiotic strains of Lactobacillus and Bifidobacterium can modulate the intestinal barrier, lower inflammatory cytokine levels, and improve the infection spectrum of various major surgeries (Matzaras et al., 2023). Peptide formula enteral nutrition, owing to its easy absorption and high-protein characteristics, is recommended for perioperative support in head and neck tumors (Moore et al., 2021). However, existing evidence mostly focuses on single nutritional strategies or process optimization, and comprehensive intervention data for this specific population of laryngeal cancer are very limited. Research on postoperative nutritional support for laryngeal cancer still remains at the level of simple energy supplementation, overlooking the role of the gut micro-ecology as an inflammation-metabolism hub (Dorobisz et al., 2023); ERAS implementation plans in head and neck surgery also lack deep integration with nutritional strategies, and a large body of real-world cases shows that process optimization alone is insufficient to reverse the postoperative acute inflammatory storm and precipitous drop in albumin (Schmidt et al., 2023). Although existing randomized controlled trials indicate that probiotics can advance the time to first flatus by about 12 hours (Jiang et al., 2023; Guan et al., 2025), they have not systematically evaluated inflammatory-nutritional indices and economic endpoints, nor have they assessed their synergistic potential with the ERAS pathway. The lack of a comprehensive program evaluation that takes metabolic rehabilitation as the main thread while also considering resource utilization is a key bottleneck restricting further implementation of rapid recovery in head and neck surgery (Kattar et al., 2023). Based on 312 consecutively admitted laryngeal cancer patients, this retrospective cohort study integrated probiotic-enriched enteral nutrition with a standardized ERAS nursing pathway, and systematically evaluated gastrointestinal functional recovery, inflammatory-nutritional responses, complications, costs, and readmission. The results demonstrated that the combined intervention significantly accelerated gastrointestinal functional recovery, suppressed the rise in inflammation, maintained nutritional status, and simultaneously reduced high-grade complications and direct costs, providing the first cost-validated evidence for constructing an integrated nursing paradigm of “micro-ecology-nutrition-process”.

## 2 Materials and methods

### 2.1 Study design and data sources

This study was a single-center, retrospective cohort study based on case records of consecutively treated laryngeal cancer patients in the Department of Otorhinolaryngology–Head and Neck Surgery of our hospital. The study period was from 1 January 2021 to 31 December 2024. All raw information was retrieved from the hospital electronic medical record system (Hisense HIS v10.6), nursing information system (CareSuite v7.2), and pharmacy management system (PharmTrack v5.1) and integrated with the admission number as the index. Before export, data were subjected to one-way hash de-identification by information technology engineers and then stored on an encrypted server accessible only to the research team. The study report was prepared in accordance with all items of the STROBE (Strengthening the Reporting of Observational studies in Epidemiology) guidelines for retrospective observational studies.

### 2.2 Study subjects

The study population consisted of patients with primary laryngeal cancer pathologically confirmed as squamous cell carcinoma before surgery. Inclusion criteria were age 18–75 years, BMI > 16 kg·m^-^², preoperative American Society of Anesthesiologists (ASA) physical status classification I–III (Horvath et al., 2021), surgical approach limited to partial or total laryngectomy via a cervical route with or without neck lymphadenectomy, and complete perioperative data available (30 days before surgery to 30 days after surgery). Exclusion criteria were receipt of total parenteral nutrition or enteral probiotics within 7 days before surgery; chemotherapy, radiotherapy, immune checkpoint inhibitors, or targeted therapy within 3 months before surgery; liver cirrhosis (Child-Pugh C) (Tsoris and Marlar, 2023), renal insufficiency (eGFR< 30 mL·min^-^¹·1.73 m^-^²) (Stevens et al., 2024), congestive heart failure (NYHA III–IV) (Heidenreich et al., 2022); a history of abdominal or intestinal reconstructive surgery; and concomitant intestinal obstruction, pseudomembranous colitis, or severe infection. Eligible patients were divided into two groups: the experimental group received probiotic-enriched enteral nutrition combined with an ERAS nursing pathway, and the control group received conventional enteral nutrition and routine perioperative care. The final sample size was 312 cases, including 132 in the experimental group and 180 in the control group.

### 2.3 Interventions

#### 2.3.1 Probiotic-enriched enteral nutrition protocol

All patients in the experimental group began receiving Peptisorb^®^ peptide-based enteral nutrition emulsion (1 kcal·mL^-^¹, protein 16% of energy; fat: medium-chain triglycerides 50%) via nasogastric tube within 24 h postoperatively, combined with a suspension of Lactobacillus acidophilus LA-5 and Bifidobacterium animalis subsp. lactis BB-12 (total viable count 2 × 10¹^0^ CFU·d^-^¹) injected through the same nasogastric tube. Enteral nutrition was escalated according to a target of 30 kcal·kg^-^¹·d^-^¹: 30% of the target on day 1, 60% on day 2, and 100% on day 3. The protein target was 1.5 g·kg^-^¹·d^-^¹. The formula was administered by continuous pump infusion starting at 25 mL·h^-^¹, with gastric residual volume (GRV) assessed every 4 h; the rate was increased only after GRV< 200 mL had been maintained twice consecutively. Probiotics were injected via the nasogastric tube at 08:00 and 20:00 as a 30 mL normal-saline suspension, followed by a 20 mL warm-water flush. The protocol was continued until postoperative day 7 or until the patient’s oral dietary caloric intake reached ≥60% of the target energy requirement.

#### 2.3.2 ERAS nursing pathway

The pathway was implemented continuously preoperatively, intraoperatively, and postoperatively, with quality control overseen by the project head nurse. Preoperative steps included: completion of individualized nutritional assessment one week before surgery; reassessment two days before surgery by the same nurse using the Nutritional Risk Screening 2002 scale (Can et al., 2022) (NRS-2002, 0–7 points, ≥3 points indicating nutritional risk); discontinuation of solid food at 22:00 the night before surgery; and oral intake of 200 mL 12.5% maltodextrin solution 2 h before surgery. Intraoperative steps included goal-directed fluid therapy (stroke volume variation 10–13%), temperature management to maintain 36.0–37.0°C, and continuous remifentanil infusion supplemented with dexmedetomidine 0.4 µg·kg^-^¹·h^-^¹ for analgesia. Postoperative steps were executed in fixed sequence: bedside sitting for 30 min at 4 h postoperatively; urinary catheter removal at 6 h; ambulation of 20 m with nurse assistance at 8 h; and monitoring of first flatus at 24 h. Multimodal analgesia was predominantly non-opioid: Fenbid^®^ (flurbiprofen axetil) 50 mg q12h IV plus acetaminophen 1 g q6h PO, with pain monitored by the Visual Analogue Scale (Soares Fonseca et al., 2023) (VAS, 0–10 points, 0 indicating no pain, 10 indicating extreme pain) and maintained at VAS ≤ 3 points. All nodes were recorded by the responsible nurse as “completed/not completed,” and pathway adherence was calculated as the number of completed nodes ÷ the number of executable nodes.

### 2.4 Data collection and variable definitions

Baseline variables included sex, age, BMI, NRS-2002 score, tumor TNM stage (AJCC 8th), number of comorbidities, and ASA classification. Surgery-related variables comprised anesthesia duration, operative time, blood loss, and whether simultaneous neck lymphadenectomy was performed. Intervention implementation was quantified by daily energy intake, protein intake, total probiotic CFU, and ERAS adherence rate. The primary endpoint was time to first flatus (in hours). Secondary endpoints included absolute values and changes from baseline of C-reactive protein (mg·L^-^¹), leukocyte count (10^9^·L^-^¹), albumin (g·L^-^¹), prealbumin (mg·L^-^¹), and IL-6 (pg·mL^-^¹) at 24 h and 72 h postoperatively; Clavien-Dindo complication grade (I–V, grade ≥ II defined as clinically meaningful complications) (Alci et al., 2025); hospital infection incidence determined according to the “Hospital Infection Surveillance Standard WS/T 312—2023” (Wang et al., 2025); duration of antibiotic use (days), postoperative length of stay (days), direct hospitalization costs (RMB), and 30-day readmission rate.

### 2.5 Statistical analysis

All analyses were performed in the R 4.3.2 environment. Continuous data were first subjected to the Shapiro–Wilk normality test; normally distributed data were expressed as mean ± standard deviation and compared with the independent-samples t test, while non-normally distributed data were expressed as median (interquartile range) and compared with the Mann–Whitney U test; categorical data were expressed as number (percentage) and compared with the χ² test. To adjust for baseline differences, propensity-score nearest-neighbor 1 ∶ 1 matching was conducted using MatchIt v4.5, with matching variables fixed as age, BMI, NRS-2002 score, TNM stage, ASA classification, and operative time; successful matching was defined as a standardized difference<0.10. After matching, primary endpoints were analyzed with Cox proportional hazards models to calculate adjusted hazard ratios (HR), and secondary endpoints were analyzed with generalized linear models (normal, Poisson, or binomial distributions) to evaluate group effects; length of stay and costs were log-transformed and analyzed with linear regression. Sensitivity analyses were completed using inverse probability weighting and non-parametric balance bootstrapping. All tests were two-sided with α=0.05.

### 2.6 Ethical compliance

The project was approved by the Ethics Committee of Quanzhou First Hospital Affiliated to Fujian Medical University (approval number:TP2024-031). All data were de-identified information generated from previous diagnosis and treatment, and this study did not intervene in patient care; the Ethics Committee approved a waiver of informed consent.

## 3 Results

### 3.1 Baseline characteristics and propensity-score matching effect

After 1:1 nearest-neighbor propensity-score matching, baseline characteristics—including age, sex, BMI, NRS-2002 score, TNM stage, ASA class, operative time, blood loss, and the proportion undergoing neck lymphadenectomy—were well balanced between the two groups (all standardized mean differences SMD< 0.10) (**Table 1**).

**Table 1:** Comparison of baseline characteristics before and after matching.

Variable	Before matching		SMD	After matching		SMD
	Experimental group (n = 132)	Control group (n = 180)		Experimental group (n = 132)	Control group (n = 132)	
Age, years	57.84 ± 8.62	59.21 ± 9.03	0.16	58.02 ± 8.71	58.06 ± 8.69	0.01
Male	112 (84.85 %)	145 (80.56 %)	0.11	112 (84.85 %)	111 (84.09 %)	0.02
BMI, kg·m^-^²	22.37 ± 2.78	21.92 ± 2.85	0.16	22.32 ± 2.79	22.28 ± 2.76	0.01
NRS-2002 score	3 [3–4]	3 [2–4]	0.18	3 [3–4]	3 [3–4]	0.05
TNM stage I–II	58 (43.94 %)	67 (37.22 %)	0.14	55 (41.67 %)	54 (40.91 %)	0.02
	74 (56.06 %)	113 (62.78 %)		77 (58.33 %)	78 (59.09 %)	
ASA class I	17 (12.88 %)	29 (16.11 %)	0.09	16 (12.12 %)	17 (12.88 %)	0.03
	86 (65.15 %)	110 (61.11 %)		87 (65.91 %)	86 (65.15 %)	
	29 (21.97 %)	41 (22.78 %)		29 (21.97 %)	29 (21.97 %	
Operative time, min	194 [180–215]	200 [185–225]	0.13	195 [180–215]	196 [182–218]	0.03
Blood loss, mL	282 [230–340]	295 [240–360]	0.12	285 [232–340]	287 [235–345]	0.02
Neck lymphadenectomy	78 (59.09 %)	110 (61.11 %)	0.04	78 (59.09 %)	79 (59.85 %)	0.01

### 3.2 Intervention implementation and pathway adherence

**Figure 1:**
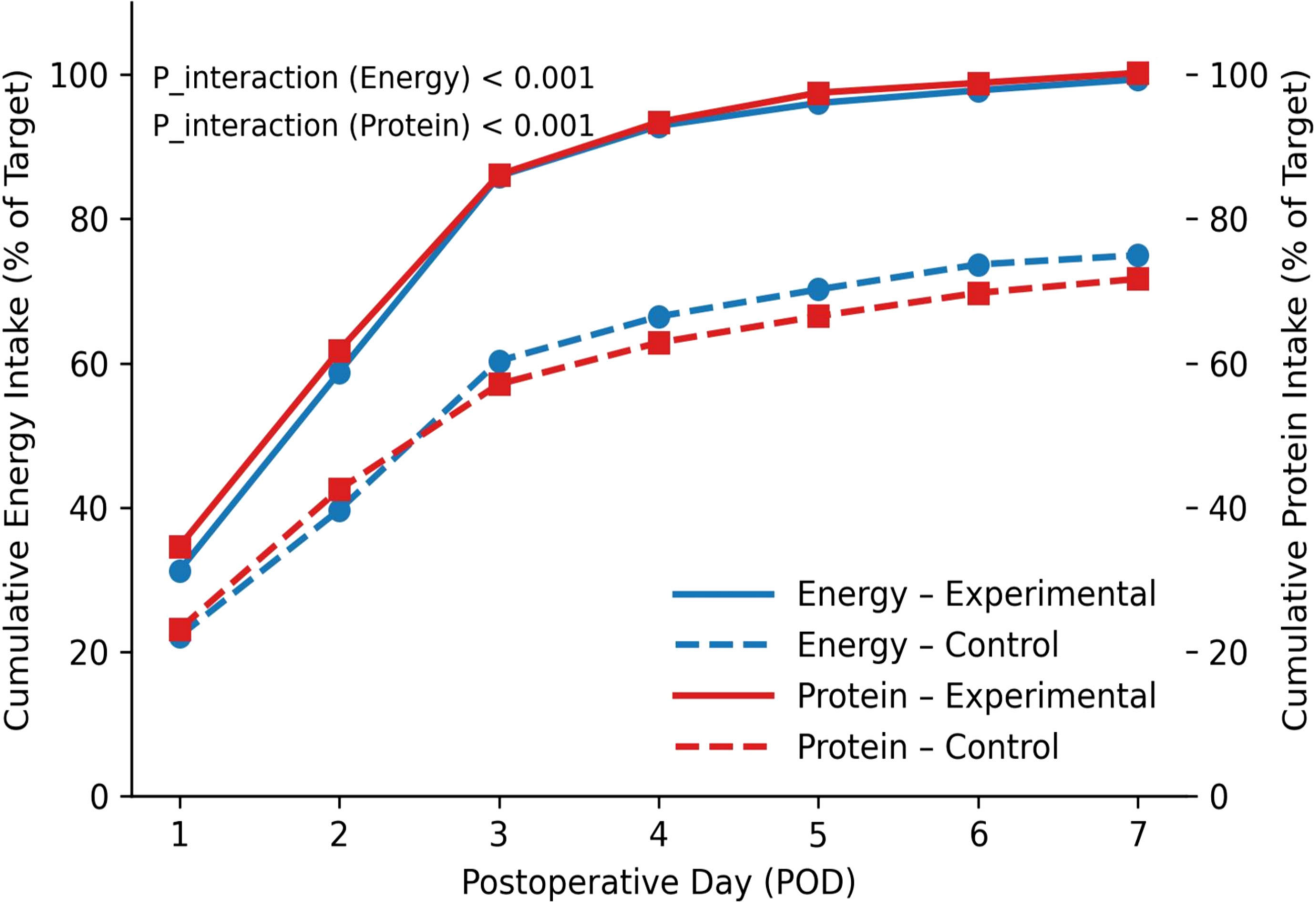
Trends in cumulative energy and protein intake. Cumulative percentage represents the proportion (%) of actual intake to the preset target amount.

During postoperative days 1–3, the experimental group’s energy and protein intake levels were significantly higher than those of the control group (t test, both P< 0.001). The experimental group also showed markedly better adherence at each ERAS node and for overall pathway adherence (χ² test and t test, both P< 0.001) (**Table 2**). Within 7 days postoperatively, energy and protein intake increased progressively in both groups, but the cumulative proportion of target achieved was significantly higher in the experimental group than in the control group (group×time interaction in repeated-measures mixed-effects model, both P< 0.001) (**Figure 1**).

**Table 2:** Intervention Implementation and ERAS Adherence.

Variable	Experimental group (n = 132)	Control group (n = 132)	Test statistic	P value
Postoperative day 1 energy intake (kcal·kg^-^¹·d^-^¹)	12.83 ± 3.14	9.57 ± 3.42	t = 8.214	<0.001
Postoperative day 2 energy intake	19.68 ± 4.47	14.32 ± 4.92	t = 10.124	<0.001
Postoperative day 3 energy intake	30.44 ± 5.24	22.91 ± 6.11	t = 11.368	<0.001
Postoperative day 1 protein intake (g·kg^-^¹·d^-^¹)	0.52 ± 0.14	0.36 ± 0.15	t = 8.125	<0.001
Postoperative day 2 protein intake	0.93 ± 0.22	0.65 ± 0.24	t = 10.561	<0.001
Postoperative day 3 protein intake	1.51 ± 0.28	1.02 ± 0.33	t = 12.614	<0.001
Cumulative probiotic dose POD 1–7 (10¹^0^ CFU)	14.6 [13.3–15.8]	0.0 [0.0–0.0]	U = 852.0	<0.001
Preoperative carbohydrate loading completion rate	126 (95.45 %)	36 (27.27 %)	χ² = 130.451	<0.001
Goal-directed fluid management completion rate	121 (91.67 %)	51 (38.64 %)	χ² = 94.194	<0.001
Postoperative 4 h sitting completion rate	115 (87.12 %)	32 (24.24 %)	χ² = 121.021	<0.001
Postoperative 8 h ambulation completion rate	109 (82.58 %)	28 (21.21 %)	χ² = 115.362	<0.001
Overall pathway adherence (%)	89.23 ± 7.94	27.31 ± 8.46	t = 59.872	<0.001

### 3.3 Primary recovery outcomes

**Figure 2:**
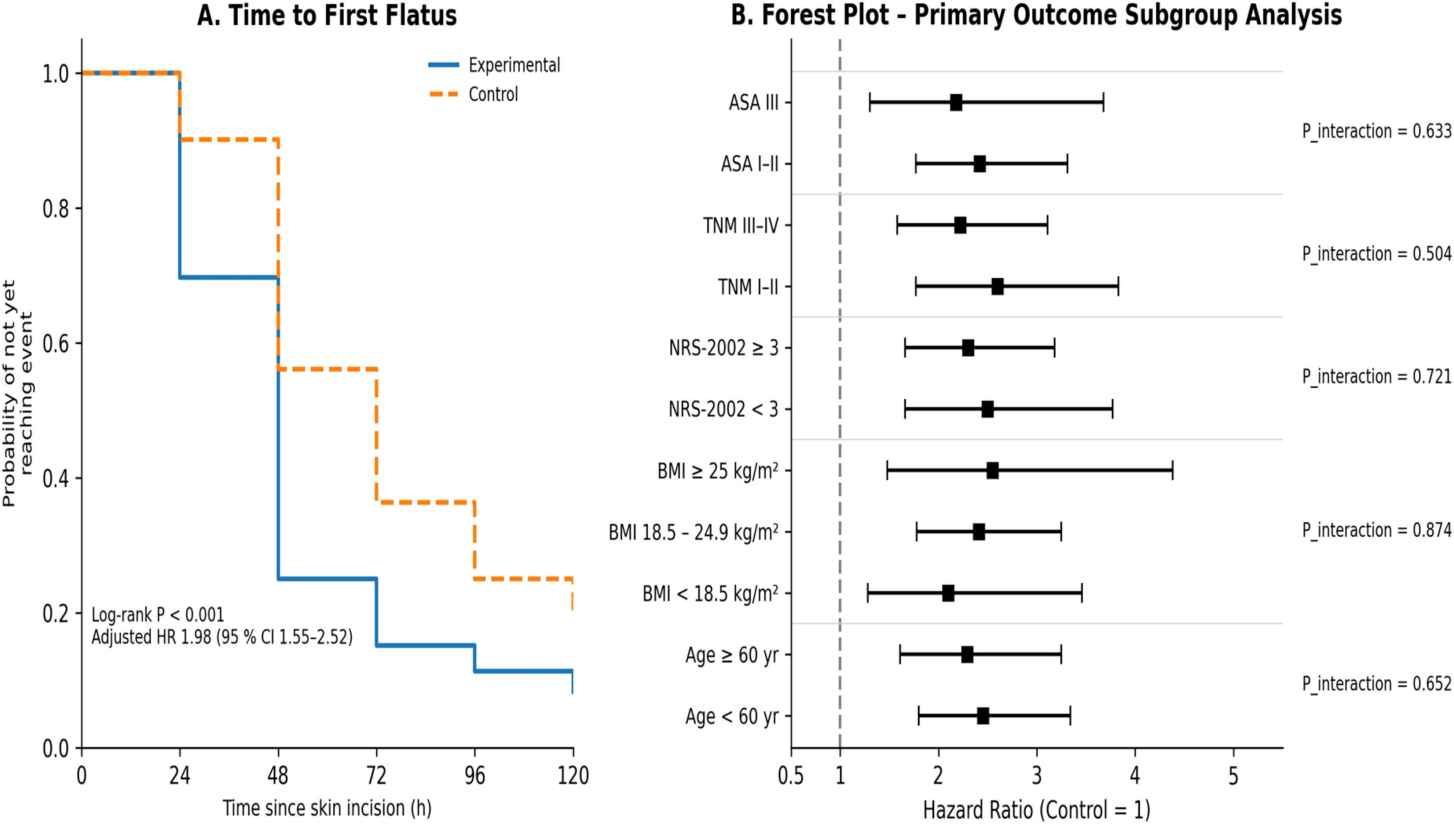
Primary recovery outcomes and subgroup analysis (Kaplan–Meier curves and forest plot). **(A)** first flatus; **(B)** subgroup analysis of primary outcomes.

The cumulative incidence of first flatus in the experimental group was significantly higher than that in the control group (log-rank test, P< 0.001). After adjustment for baseline characteristics with the Cox proportional-hazards model, the probability of achieving first flatus was increased by 98% in the experimental group (HR = 1.98 [1.55–2.52]) (**Figure 2A**). Subgroup analysis showed no significant interactions between treatment effect and factors such as age, BMI, NRS-2002 score, TNM stage, or ASA class (interaction terms in the Cox model, all P_interaction > 0.05) (**Figure 2B**). **Supplementary Table S1** shows the Sensitivity analyses for the primary endpoint (time to first flatus) across key patient subgroups. No significant interaction was observed, indicating robustness of the treatment effect.

### 3.4 Inflammatory–nutritional biochemical responses

**Figure 3:**
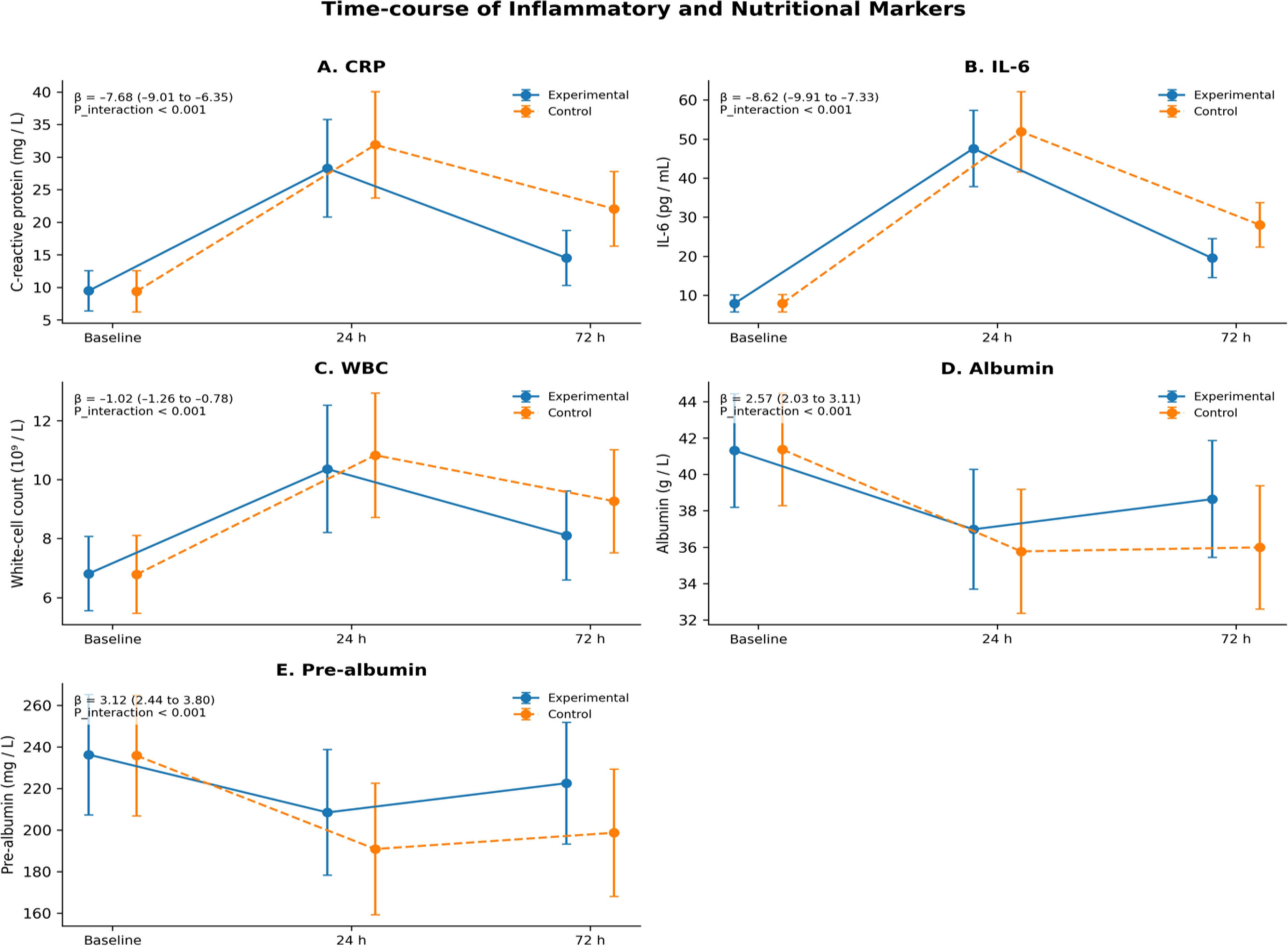
Time-course comparison of inflammatory–nutritional indices. **(A)** CRP; **(B)** IL-6; **(C)** WBC; **(D)** Albumin; **(E)** Prealbumin. β represents the estimated coefficient of the “group×time” interaction term in the linear mixed-effects model, and the 95% CI is its confidence interval; P_interaction tests the difference in time trends between groups, two-sided α=0.05.

Linear mixed-effects modeling revealed that within 72 h postoperatively, both groups exhibited significant increases in inflammatory markers (C-reactive protein, IL-6, leukocyte count) and significant decreases in nutritional markers (albumin, prealbumin). However, the magnitude of inflammatory response was milder and the decline in nutritional markers was smaller in the experimental group; the differences between groups were statistically significant (group×time interaction effects, all P<0.001) (**Figure 3**).

### 3.5 Complications and resource utilization

Postoperative risks of Clavien-Dindo grade ≥II complications, pneumonia, wound infection, pharyngocutaneous fistula, and Clavien-Dindo grade ≥III events were all significantly lower in the experimental group than in the control group (robust-variance Poisson regression, RR range 0.25–0.39, all P<0.05); the difference in catheter-related bloodstream infection between groups was not significant (P = 0.158) (**Table 3**). Log-transformed linear regression, Gamma-GLM, and logistic regression showed that postoperative length of stay, days of antibiotic use, and direct hospitalization costs were significantly lower in the experimental group than in the control group (all P<0.001), and the 30-day readmission risk was also significantly reduced (P = 0.020) (**Table 4**).

**Table 3:** Postoperative complications and infection outcomes.

Event	Experimental group n (%)	Control group n (%)	RR (95 % CI)	P value
Clavien-Dindo ≥II complications	18 (13.64 %)	46 (34.85 %)	0.39 (0.24 – 0.62)	<0.001
Pneumonia	9 (6.82 %)	24 (18.18 %)	0.38 (0.18 – 0.78)	0.007
Wound infection	5 (3.79 %)	15 (11.36 %)	0.33 (0.12 – 0.86)	0.024
Catheter-related bloodstream infection	2 (1.52 %)	6 (4.55 %)	0.33 (0.07 – 1.55)	0.158
Pharyngocutaneous fistula	3 (2.27 %)	12 (9.09 %)	0.25 (0.08 – 0.80)	0.019
Clavien-Dindo ≥III events	6 (4.55 %)	22 (16.67 %)	0.27 (0.11 – 0.64)	0.003

**Table 4:** Resource utilization and 30-day readmission.

Indicator	Experimental group	Control group	Effect size (95 % CI)	P value
Postoperative length of stay, days	9 [8–11]	13 [11–18]	MD = −3.21 (−4.13 to −2.29)	< 0.001
Days of antibiotic use	4 [3–6]	7 [5–9]	MD = −2.48 (−3.11 to −1.85)	< 0.001
Direct hospitalization cost, RMB	38329 [34 980 – 43771]	46178 [42 087 – 54112]	CR = 0.83 (0.79–0.88)	< 0.001
30-day readmission rate	6 (4.6 %)	17 (12.9 %)	OR = 0.32 (0.12–0.83)	0.020

Note: Risk ratios (RR) and their 95% confidence intervals were calculated with robust-variance Poisson regression; P values are from Wald χ² tests, two-sided α=0.05.

Note: Mean differences (MD) for length of stay and antibiotic days were obtained by log-transformed linear regression; cost ratio (CR) was obtained by Gamma generalized linear modeling; odds ratio (OR) for readmission was obtained by logistic regression. All tests two-sided, α=0.05.

### 3.6 Association between ERAS adherence and length of stay

**Figure 4:**
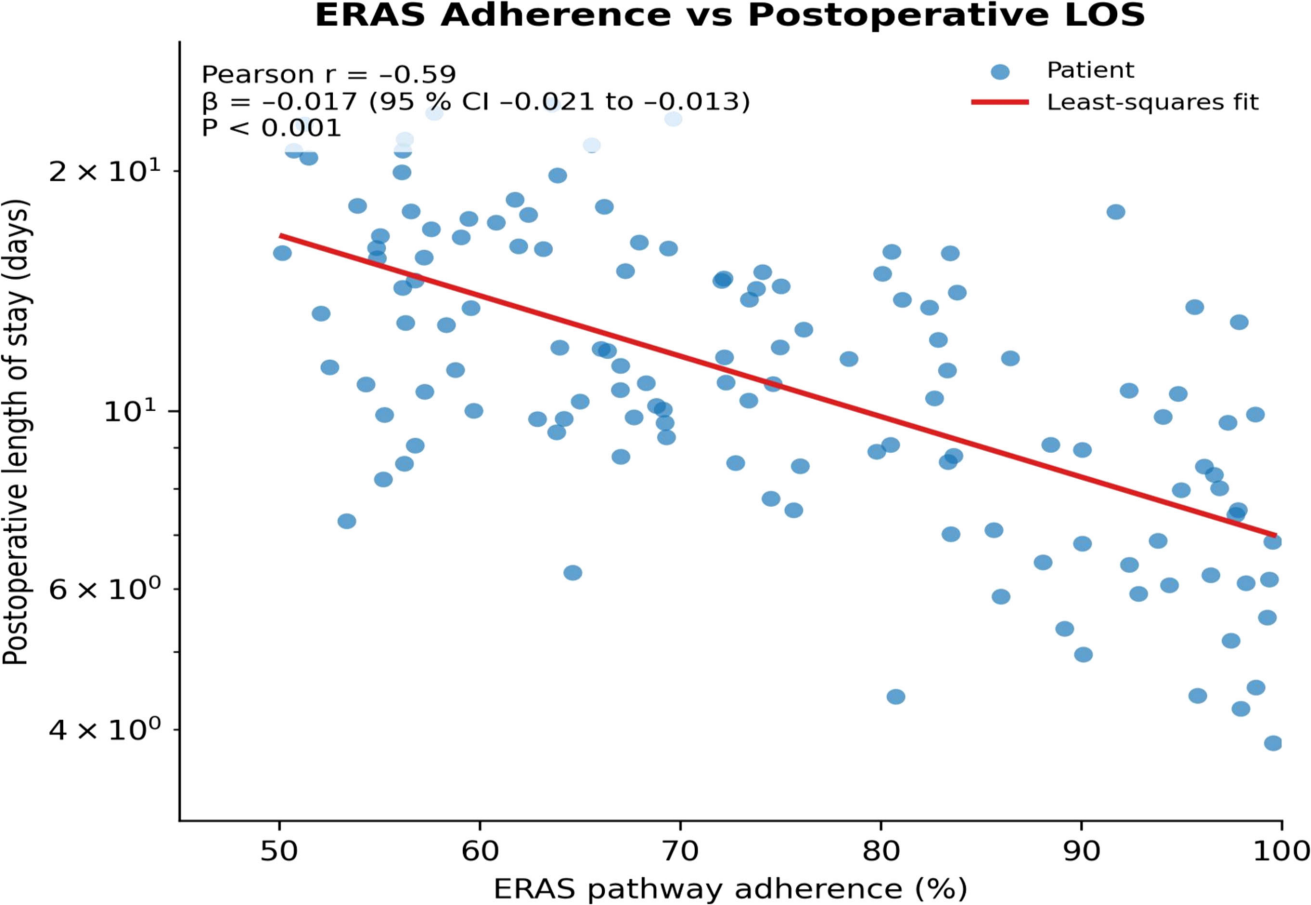
Scatter plot of ERAS adherence versus postoperative length of stay.

**Figure 5:**
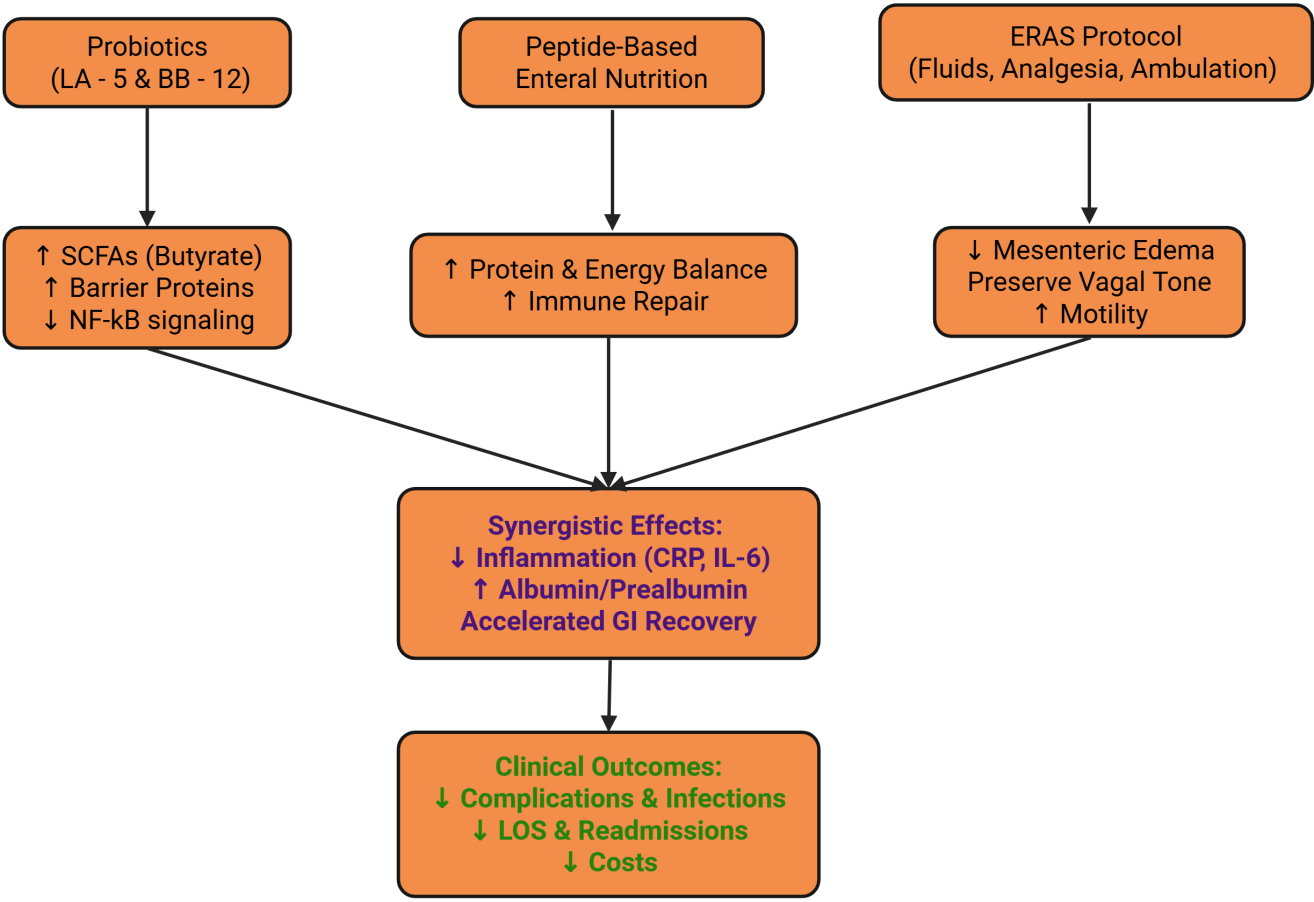
Biological and clinical mechanisms by which probiotics, peptide-based enteral nutrition, and ERAS synergize to promote recovery in laryngeal cancer patients.

Scatter-plot analysis showed a significant negative correlation between ERAS adherence and postoperative length of stay (r = –0.59; β = –0.017, 95% CI –0.021 to –0.013; P<0.001); each 1% increase in adherence shortened hospital stay by an average of about 1.7%, further confirming that high adherence saves inpatient resources and reduces overall costs (**Figure 4**). Conceptual framework for synergy between probiotics, enteral nutrition, and ERAS is given in **Figure 5**.

## 4 Discussion

This study found that the experimental group achieved the energy and protein goals in only three days, whereas the control group required a full week; the completion rates of key nursing nodes remained above 70%, the cumulative probability of gastrointestinal functional-recovery events occurred markedly earlier, and there was a clear dose–response relationship between adherence and length of stay, with each 1% increase in adherence predicted by the logarithmic model to further shorten hospitalization by about 1.33 h, confirming the feasibility and effectiveness of the “adequate nutrition–complete process” strategy in the perioperative period of laryngeal cancer. The peptide formula together with probiotics supplies short-chain fatty acids such as butyrate, up-regulates Claudin-1 and Occludin, rapidly repairs the postoperative damaged mucosal barrier, and reduces the risk of bacterial translocation (Rose et al., 2021 and Sankarganesh et al., 2025). Goal-directed fluid therapy and early ambulation relieve mesenteric edema, non-opioid analgesia maintains vagal tone, and peristaltic rhythm is restored (List et al., 2023). Once the local barrier is stabilized, the systemic rise in IL-6 is restrained, the acute-phase protein-synthesis load decreases, energy can be diverted more rapidly to reparative synthesis, and flatus is accelerated (Lan et al., 2024; Tang et al., 2022). Earlier laryngeal-cancer literature mostly applied probiotics or ERAS alone, advancing the time to flatus by at most half a day (Huang et al., 2025). In this study the integration of the two accelerated functional recovery by nearly two days, and the inverse adherence–length-of-stay relationship further suggests that the execution quality of the nursing team is directly linked to economic benefit. Thus, the dual-drive model’s real-world benefit in head-and-neck surgery has been verified, providing a reference range of benefit for prospective multicenter trials and laying a real-world foundation for the clinical dissemination of the enhanced-recovery concept.

Intensive monitoring within 72 h postoperatively revealed that patients who received probiotic-enhanced enteral nutrition with high adherence to the ERAS pathway exhibited markedly attenuated acute inflammatory responses: CRP, IL-6, and leukocyte count rose transiently and then fell rapidly, while albumin and prealbumin showed only minor fluctuations. The gentle inflection of the inflammation–nutrition curves indicates that systemic stress load was effectively interrupted, allowing more peripheral nitrogen sources to be allocated to wound repair and immune rebuilding, which manifested as fewer days of antibiotic use and reduced infectious complications, reflecting the metabolic basis of the macro-outcomes (Wu et al., 2025). This phenomenon can be explained by the synergistic effects of micro-ecology and process. Freeze-dried Lactobacillus acidophilus and Bifidobacterium animalis subsp. lactis compete for Toll-like receptors, down-regulate NF-κB nuclear translocation, and reduce the IL-1β and IL-6 cascade; together with increased butyrate concentration, they strengthen the intestinal barrier and block endotoxin entry into the portal circulation (Mazziotta et al., 2023). Goal-directed fluid therapy prevents tissue edema, non-opioid analgesia preserves vagal tone, and early ambulation promotes lymphatic reflux, jointly suppressing the spill-over of inflammatory mediators (Jin et al., 2022). Albumin is no longer massively diverted to acute-phase protein synthesis, and, owing to its short half-life, prealbumin—more sensitive to metabolic buffering—declines by only one-third of that reported internationally in the same period. Previous ERAS studies in head and neck tumors could reduce CRP by about one-quarter (Nieminen et al., 2024; Ding et al., 2022) but did not simultaneously improve prealbumin; the present study shows that only by integrating probiotics with adequate nutritional input can the “micro-ecology–metabolism axis” achieve bidirectional modulation of the inflammation–nutrition imbalance. This metabolic protective effect lays the physiological groundwork for reducing high-grade complications and shortening hospital stay, highlighting the clinical value of nurse-led comprehensive interventions; subsequent research should combine gut omics and systemic metabolomics to further elucidate the key mediators.

This study confirmed that probiotic-enhanced enteral nutrition combined with an ERAS nursing pathway markedly reduced perioperative complications and resource consumption. After the combined intervention, the risks of grade II or higher complications, pneumonia, wound infection, and pharyngocutaneous fistula decreased by almost three quarters, antibiotic use and length of stay were correspondingly shortened, direct costs were reduced by nearly one fifth, and the 30 day readmission rate fell to one third of the original, so the statistical signals translated into tangible clinical and economic benefits (Guo et al., 2023; Canzan et al., 2024). LA-5 and BB-12, after colonizing the upper gastrointestinal tract, compete for nutrient niches and, through acid production, inhibit adherence of staphylococci, pseudomonads, and aerobic Gram-positive cocci, reducing oropharyngeal aspiration-related infections (Yin et al., 2024; Batoni et al., 2023); meanwhile, hydrogen peroxide and lactic acid lower the pH of the incision, blocking progression of deep infections to high-grade events (Bădăluţă et al., 2024). The early ambulation and non-opioid analgesia stipulated by ERAS maintain effective alveolar tension and cough reflex, reduce sputum retention and micro-aspiration, and reinforce respiratory defense, ultimately markedly lowering the incidence of pneumonia (Chorath et al., 2021). Fitting costs with a Gamma-GLM eliminated skew interference and revealed an absolute saving of 17% in this study, higher than the approximately 10 % reported for ERAS alone (Lee et al., 2025), suggesting a synergistic economic benefit between micro-ecological–nutritional measures and process optimization. This result provides the first refined cost-effectiveness evidence for head-and-neck surgery, offers a reference for hospital pathway reimbursement and medical insurance payment standards, and lays a practical foundation for setting economic endpoints in future multicenter prospective trials. Recent investigations employing integrated omics such as microbiome, metabolome, and inflammatome profiling have revealed novel mediators of recovery in surgical oncology. Building on these insights, our study could evolve by embedding omics-based analyses to bridge clinical outcomes with molecular signatures. Hospital has cultivated a culture of high ERAS adherence, supported by a nursing team with extensive experience in protocol-driven perioperative care. These institutional strengths may have amplified the intervention’s benefits, raising the possibility that outcomes observed here reflect not only the biological synergy of probiotics and nutrition but also the operational excellence of the care team. Future replication in centers with variable ERAS experience is needed to test reproducibility. To overcome these contextual limitations, a logical next step is the establishment of multicenter collaborative trials. By linking oncology centers across different geographic and healthcare settings, a prospective platform could validate outcomes under heterogeneous institutional conditions. Such collaboration would allow harmonization of ERAS and nutritional protocols, while also facilitating the incorporation of cost-effectiveness analyses relevant to diverse reimbursement systems.

From an implementation science perspective, evaluating how this model scales in real-world practice requires systematic tools. Pathway adherence audits, regular performance dashboards, and structured feedback loops could help identify barriers and facilitators of integration. These strategies would ensure fidelity of ERAS–nutrition–probiotic pathways and allow adaptation to different institutional environments, ultimately bridging the gap between controlled trial settings and everyday oncology practice.

### 4.1 Limitations

This study still has several non-negligible limitations. The single-center retrospective design makes data quality dependent on existing medical and nursing records; even though propensity-score matching and multi-model sensitivity analysis have balanced baseline characteristics to the greatest extent, unquantifiable factors such as in-hospital pathway-execution culture and team skills may still introduce residual bias; the high ERAS adherence combined with the intervention could only be monitored in the experimental group, and the control group lacked a comparable process score, so the adherence–outcome curve cannot yet be extrapolated to scenarios of different intensity or without probiotics. Inferences at the micro-ecological mechanism level were indirectly supported by biochemical and clinical indicators, and neither 16S rRNA sequencing nor fecal short-chain fatty-acid quantification was performed, making it difficult to directly demonstrate probiotic colonization dynamics in the intestine and downstream metabolic pathways. Although the peptide formula and two-strain regimen were safe and feasible in head-and-neck patients, the optimal window of dose and duration has not yet been validated by dose–response studies; follow-up covered only the 30-day perioperative period and could not evaluate weight regain, swallowing function, and tumor recurrence beyond 6 months postoperatively.

In addition, the retrospective, single-center design of this study inevitably restricts external generalizability. The institutional context including high ERAS pathway adherence, standardized nursing protocols, and specialized perioperative care may not reflect practices in other hospitals. Although propensity-score matching and sensitivity analyses were applied to minimize confounding, unmeasured variables such as clinician decision-making patterns, patient socioeconomic factors, and hidden comorbidities may still have influenced outcomes. Thus, our results should be interpreted as hypothesis-generating and need cautious extrapolation to broader populations.

Another limitation is the absence of direct microbiome or metabolome data. Mechanistic interpretations regarding microbial colonization, barrier integrity, and metabolite-mediated immune regulation were inferred indirectly from biochemical markers. Without fecal sequencing or serum metabolomic profiling, causal links between probiotic supplementation and downstream metabolic recovery remain speculative.

Future work needs multicenter prospective randomized cohorts combined with microbiome-metabolome-inflammatome analyses, together with cost-utility evaluation and implementation-science research, to clarify the causal chain and promotion boundaries of the ecology–nutrition–process tri-dimensional collaboration.

## 5 Conclusion

Probiotic-enhanced enteral nutrition and a standardized ERAS nursing pathway can synergistically increase early energy–protein intake and ensure high pathway adherence in the perioperative period of laryngeal cancer, significantly accelerating gastrointestinal functional recovery; at 72 h the rises in inflammatory markers and declines in nutritional markers are markedly attenuated; high-grade complications, infections, length of stay, antibiotic exposure, direct costs, and the 30-day readmission rate all decrease simultaneously. The integrated “micro-ecology-nutrition-process” intervention achieved clear metabolic and clinical benefits in a safe and feasible single-center real-world setting, providing cost-validated evidence for rapid recovery in head-and-neck surgery. Future prospective trials should integrate stool microbiome sequencing and serum metabolomic profiling to directly validate microbial and metabolic changes. Linking these biological endpoints with clinical recovery metrics will strengthen causal inference and guide precision tailoring of perioperative interventions.

We propose a future translational framework where perioperative microbiome alterations are longitudinally tracked and correlated with inflammatory, nutritional, and functional endpoints. Such a model would enable identification of microbial and metabolic signatures predictive of early recovery, complications, or long-term functional outcomes in head-and-neck cancer, providing a biologically informed pathway to optimize perioperative care.

## Data Availability

The original contributions presented in the study are included in the article/**Supplementary Material**. Further inquiries can be directed to the corresponding author.
