# Supplementary material for: Impact of probiotic-enriched enteral nutrition combined with an ERAS protocol on postoperative recovery and metabolic rehabilitation in laryngeal cancer patients: a single-center retrospective cohort study
Source: Front Cell Infect Microbiol. 2025 Nov 3;15:1692767. doi: 10.3389/fcimb.2025.1692767 (PMC12620478; doi:10.3389/fcimb.2025.1692767)
Supplement: Supplementary file 1 [file Table1.docx]

| Subgroup | HR for First Flatus (95% CI) | P for Interaction |
| --- | --- | --- |
| Age (<60 vs ≥60 years) | 1.94 (1.42–2.64) vs 2.01 (1.49–2.71) | 0.72 |
| BMI (<22 vs ≥22 kg·m²) | 1.87 (1.39–2.55) vs 2.06 (1.51–2.80) | 0.68 |
| TNM Stage (I–II vs III–IV) | 2.02 (1.48–2.76) vs 1.95 (1.47–2.61) | 0.81 |
| ASA Class (I–II vs III) | 1.99 (1.52–2.62) vs 1.91 (1.40–2.60) | 0.84 |

**Table S1: Sensitivity analysis**
